# Structural, Biochemical, and Biophysical Characterization of Idelalisib Binding to Phosphoinositide 3-Kinase δ*

**DOI:** 10.1074/jbc.M114.634683

**Published:** 2015-01-28

**Authors:** John R. Somoza, David Koditek, Armando G. Villaseñor, Nikolai Novikov, Melanie H. Wong, Albert Liclican, Weimei Xing, Leanna Lagpacan, Ruth Wang, Brian E. Schultz, Giuseppe A. Papalia, Dharmaraj Samuel, Latesh Lad, Mary E. McGrath

**Affiliations:** From the Departments of ‡ Structural Chemistry and; § Biology, Gilead Sciences, Inc., Foster City, California 94404

**Keywords:** Enzyme Kinetics, Enzyme Mechanism, Enzyme Purification, Phosphatidylinositide 3-Kinase (PI 3-Kinase), Surface Plasmon Resonance (SPR), CAL-101, GS-1101, PI3K, PI3Kδ, Zydelig

## Abstract

Idelalisib (also known as GS-1101, CAL-101, IC489666, and Zydelig) is a PI3Kδ inhibitor that has recently been approved for the treatment of several hematological malignancies. Given its use in human diseases, we needed a clear picture of how idelalisib binds to and inhibits PI3Kδ. Our data show that idelalisib is a potent and selective inhibitor of the kinase activity of PI3Kδ. A kinetic characterization clearly demonstrated ATP-competitive inhibition, and several additional biochemical and biophysical assays showed that the compound binds reversibly and noncovalently to the kinase. A crystal structure of idelalisib bound to the p110δ subunit of PI3Kδ furthers our understanding of the binding interactions that confer the potency and selectivity of idelalisib.

## Teaser

**Background:** Idelalisib is a PI3Kδ inhibitor used to treat hematological malignancies.

**Results:** Idelalisib is selective, noncovalent, reversible, and ATP-competitive.

**Conclusion:** The crystal structure helps explain the potency and selectivity of idelalisib. The biophysical and biochemical data clarify the details of the inhibitor's interactions with PI3Kδ.

**Significance:** Its use in humans makes it important to understand how idelalisib inhibits PI3Kδ.

## Introduction

Class I PI3Ks catalyze the intracellular conversion of phosphatidylinositol 4,5-bisphosphate (PIP2)^2^ to phosphatidylinositol 3,4,5-trisphosphate (PIP3), which functions as a second messenger, recruiting and activating signaling proteins such as Akt. Signals are relayed from receptor tyrosine kinases and G protein-coupled receptors to pathways regulating metabolism, cell growth and proliferation, motility, and differentiation.

PI3Kδ is a class I PI3K that is formed by a catalytic subunit (p110δ) and a regulatory subunit (p85). The observation that PI3Kδ is selectively expressed in leukocytes suggested that this isoform might be a therapeutic target for diseases in which there is pathological activation of the Akt pathway in hematopoietic cells. Hematological malignancies of B cells, including indolent non-Hodgkin lymphoma, chronic lymphocytic leukemia, and mantle cell lymphoma, have constitutively active PI3K/Akt signaling pathways and respond to pathway inhibition (1–4).

Aberrant signaling in B cells is also found in a number of inflammatory diseases. PI3Kδ inhibition interferes with B cell activation, survival, and migration (5). In rheumatoid arthritis, PI3Kδ is highly expressed in the rheumatoid arthritis synovium (6) and in fibroblast-like synoviocytes, macrophages, and Th1 and Th17 cells (7). PI3Kδ inhibition can modulate both B cell and T cell production of inflammatory cytokines. In models of rheumatoid arthritis, PI3Kδ inhibitors have reduced inflammation and decreased bone and cartilage erosion (8).

Additional inflammatory diseases may benefit from blocking PI3Kδ activity. Excessive PI3Kδ activity in mast cells (9), neutrophils (10), T cells (11), eosinophils, and B cells contributes to the pathogenesis of allergic asthma. In a mouse model of asthma, PI3Kδ inhibition attenuated airway hyper-responsiveness; decreased the influx of neutrophils, eosinophils, and lymphocytes to airways; and reduced IL-17 production (12).

**FIGURE 1.**
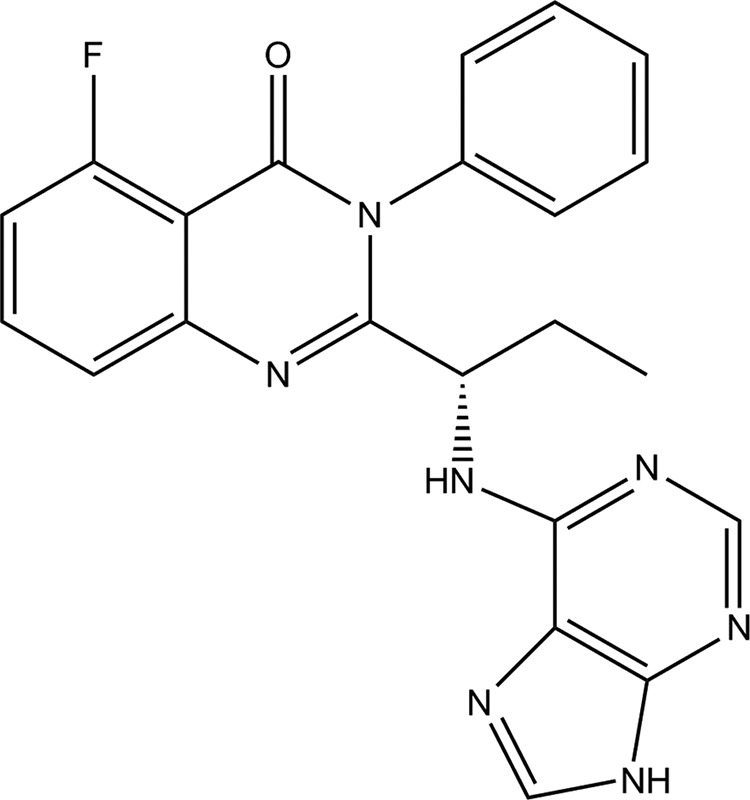
**The structure of idelalisib (5-fluoro-3-phenyl-2-[(1S)-1-(9H-purin-6-ylamino)propyl]quinazolin-4(3H)-one).**

Idelalisib is a potent and selective inhibitor of the kinase activity of PI3Kδ (Fig. 1). The efficacy of this compound has been demonstrated in a series of human clinical studies (13–17), which led to the recent approval of Zydelig (idelalisib) in the United States and European Union. In the United States, Zydelig is indicated, in combination with rituximab, for the treatment of patients with relapsed chronic lymphocytic leukemia and as a monotherapy for patients with relapsed follicular B cell non-Hodgkin lymphoma and relapsed small lymphocytic lymphoma (18). In the European Union, Zydelig is indicated, in combination with rituximab, for the treatment of patients with relapsed chronic lymphocytic leukemia and refractory follicular B cell non-Hodgkin lymphoma and as first line therapy in chronic lymphocytic leukemia patients with 17p deletion or TP53 mutation who are unsuitable for chemoimmunotherapy (19). In this study, we describe the results of a set of biochemical and biophysical experiments aimed at defining how idelalisib inhibits PI3Kδ.

## EXPERIMENTAL PROCEDURES

### 

#### 

##### Protein Expression and Purification

The human p110α and murine p110δ catalytic subunits were co-expressed with the p85α regulatory subunit and purified as heterodimers. Human p110γ was expressed and purified without a regulatory subunit. PI3Kβ was purchased from Millipore (Billerica, MA) (item 14-603, lot 1994929-B).

Polyhistidine tags engineered at the N termini of the p110 constructs were used to purify the protein. The p110 domains and p85α were cloned into *pFastBacHTa* and *pFastBac* dual vectors, respectively. *Spodoptera frugiperda* (*Sf21*) cells at a density of 1.5 × 10^6^ cells/ml were infected with an optimized ratio of viruses for 70 h at 27 °C. 20 g of cell pellets were processed for each of these samples, and the protein was purified using a Ni-NTA FF column (GE Life Sciences) and was buffer-exchanged using a KW2003 SEC column (Showa Denko America, New York, NY). The final sample of p110α-p85α was delivered in 20 mm HEPES (pH 7.5), 150 mm NaCl, and 1 mm DTT. P110δ-p85α was delivered in 25 mm HEPES (pH 7.5), 40% glycerol, 300 mm NaCl, and 3 mm CHAPS. P110γ was delivered in 25 mm HEPES (pH 7.5), 10% glycerol, 200 mm NaCl, and 2 mm DTT.

A second p110δ construct was prepared with an rTEV-digestible site inserted after the adaptor-binding domain (ABD) and co-expressed with the iSH2 domain of p85α. The cloning, expression, and purification strategies for this construct were adapted from a published protocol (20). The addition of 3 mm CHAPS to the lysis buffer helped maximize the recovery of the p110δ/iSH2-p85α complex from the cell pellet. Cell lysate after clarification was loaded on a Ni-NTA FF column and extensively washed with lysis buffer (20 CV) containing 1 m NaCl to remove contaminant proteins. The pure heterodimer was eluted from the column and digested with the rTEV protease to remove the iSH2 and ABD domains. The resulting protein was passed through a Ni-NTA FF column, and the flow-through portion containing ΔABD-p110δ protein was pooled, concentrated, and loaded onto a size exclusion chromatography column (KW2003) pre-equilibrated with a buffer containing 20 mm Tris-HCl (pH 7.2), 50 mm (NH_4_)_2_SO_4_, 1% (v/v) ethylene glycol, 1% (w/v) betaine, 300 mm CHAPS, and 5 mm tris(2-carboxyethyl)phosphine hydrochloride (TCEP). Fractions containing the protein of interest were pooled and concentrated to 20 mg/ml for crystallization trials. The final sample had 15% polydispersity as measured by dynamic light scattering (Wyatt Technology, Santa Barbara, CA), and the purity was 98% as determined by SDS-PAGE analysis.

##### Enzymatic Activity of the Class I PI3K Isoforms

We measured IC_50_ values for all four class I PI3Ks. The full-length protein used for the PI3Kα, γ, and δ measurements is described above. The PI3Kβ consisted of a complex of N-terminal His_6_-tagged recombinant full-length human p110β and untagged, full-length, human p85α (Millipore).

The enzymatic activity of the class I PI3K isoforms was measured using a time-resolved FRET assay that monitors formation of the product PIP3, because it competes with fluorescently labeled PIP3 for binding to the GRP-1 pleckstrin homology domain protein. An increase in PIP3 product results in a decrease in time-resolved FRET signal as the labeled fluorophore is displaced from the GRP-1. PI3K isoforms were assayed under initial rate conditions in the presence of 25 mm HEPES (pH 7.4), and 2 × *K_m_* ATP (100–300 μm), 10 μm PIP2, 5% glycerol, 5 mm MgCl_2_, 50 mm NaCl, 0.05% (v/v) CHAPS, 1 mm dithiothreitol, 1% (v/v) DMSO at the following concentrations for each isoform: PI3Kα, β, δ at 25–50 pm and PI3Kγ at 2 nm. After an assay reaction time of 30 min at 25 °C, reactions were terminated with a final concentration of 10 mm EDTA, 10 nm labeled PIP3, and 35 nm europium-labeled GRP-1 detector protein before reading time-resolved FRET on an Envision plate reader (excitation, 340 nm; emission, 615/665 nm; delay, 100 μs; and read window, 500 μs). The data were normalized based on positive (1 μm wortmannin) and negative (DMSO) controls, and IC_50_ values were calculated from the fit of the dose-response curves to a four-parameter equation. All IC_50_ values represent geometric mean values of a minimum of four determinations. These assays generally produced results within 3-fold of the reported mean. The KINOMEscan platform (DiscoveRx, San Diego, CA) was used to assess the interaction of idelalisib, at a concentration of 10 μm, with the ATP-binding site of a broad set of kinases (21).

##### Competition of Idelalisib with ATP

To solutions of recombinant PI3Kδ (p110δ-p85α) (final concentration, 15 pm) in reaction buffer from the PI3K HTRF assay kit (Millipore) was added idelalisib at concentrations from 0 to 80 nm (final concentration). After a 1-h preincubation period, PIP2 was added to a final concentration of 10 μm, and ATP was added at concentrations from 0 to 1.5 mm to initiate the reaction. After 45 min, the reactions were quenched with a stop solution from the assay kit. A detection solution was then added to each well, and the mixture was further incubated for 2 h. Time-resolved fluorescence was measured with a Tecan Infinite M1000 plate reader, using an excitation wavelength of 330 nm and emission wavelengths of 620 and 665 nm. The ratio of emission at 665 nm to that at 620 nm was used as the measure of reaction rate. The rate data were fit globally using the equation *v* = *V*_max_ [S]/(*K_m_*(1 + [I]/*K_i_*) + [S](1 + [I]/α*K_i_*)), where *v* is the observed reaction rate, *V*_max_ is the maximum reaction rate, [S] is the concentration of ATP, *K_m_* is the Michaelis constant for ATP, [I] is the concentration of idelalisib, *K_i_* is the inhibition constant for idelalisib, and α is a multiplier for *K_i_* that describes the competition behavior. Values of α ≫ 1 are indicative of competitive inhibition, values of α approximately equal to 1 imply noncompetitive inhibition, and values of α ≪ 1 are characteristic of uncompetitive inhibitors (22).

##### Reversibility Assay

Solutions of 3 nm PI3Kδ-p 85α were preincubated with either 400 nm idelalisib or 100 nm wortmannin for 1 h. One microliter of solution was transferred to 99 μl of reaction buffer solution containing 10 μm PIP2 and 300 μm ATP to initiate the enzyme reaction. At 5-min intervals, samples from each reaction were quenched with HTRF stop solution, and fluorescence was measured using the detection kit as described above. The reactions were measured over a time frame of 40 min. Control reactions consisted of the reaction of 30 pm PI3Kδ in the absence of inhibitor, the reaction of 30 pm PI3Kδ in the presence of 400 nm idelalisib or 100 nm wortmannin, and the reaction of 30 pm PI3Kδ in the presence of 4 nm idelalisib or 1 nm wortmannin. The data were analyzed with linear least square fits to obtain reaction rates.

##### Surface Plasmon Resonance Binding Assay

ΔABD-p110δ was minimally biotinylated using a 1:1 molar ratio of protein to EZ-link sulfo-NHS-LC-LC-biotin (ThermoScientific; catalog no. 21338). The binding site was protected from biotinylation by adding 10 μm of idelalisib to 9 μm of protein prior to addition of EZ-link sulfo-NHS-LC-LC-biotin. Biotinylation in the absence of idelalisib protection was also tested. Following incubation for 1 h at 4 °C, the mixture was desalted using a Zeba spin desalting column (ThermoScientific; catalog no. 89883) pre-equilibrated in Biacore running buffer (25 mm HEPES, pH 7.5, 150 mm NaCl, 5 mm MgCl_2_, 1 mm TCEP, 5% glycerol, 0.05% P20) to remove any unreacted biotin. The presence of one biotin molecule per protein molecule was confirmed by mass spectrometry. Characterization of inhibitor binding and dissociation was performed using a Biacore T100 instrument and research grade series S CM5 sensor chips (GE Healthcare; catalog no. BR-1005-30). Before use, CM5 sensor chips were preconditioned using two 6-s pulses each of 100 mm HCl, 50 mm NaOH, 0.5% (w/v) SDS, and deionized H_2_O at a flow rate of 100 μl/min. Approximately 15,000 response units (RU) of neutravidin was immobilized on all four surfaces via standard amine-coupling chemistry (GE Healthcare; catalog no. BR-1000-50) in HBS-P buffer (10 mm HEPES, pH 7.4, 150 mm NaCl, 0.005% P20). Biotinylated protein was injected over separate flow cells to achieve capture levels ranging between 6,000 and 14,000 response units. Any remaining free sites on neutravidin were blocked with two 5-min pulses of 50 μm EZ-Link-Amine-PEG_2_-Biotin (ThermoScientific; catalog no. 21346) at a flow rate of 30 μl/min. Idelalisib at concentrations of 0.12, 0.37, 1.1, 3.3, 10, and 30 nm (in duplicate) was injected for a 90-s association time and a 1–200-s dissociation time at a flow rate of 100 μl/min. Wortmannin was injected on a separate sensor chip at a concentration of 2 μm for an 8-min association phase and a 1-hour dissociation phase. At the end of each injection cycle, the injection needle and tubing were washed with 50% DMSO using the “extra wash” command. A five-point DMSO concentration series (ranging from 0.8% to 1.2% DMSO) was included to generate a calibration curve to correct for excluded volume effects. Sensorgrams were double-referenced and corrected for solvent effects using Scrubber 2.0c software (Biologic Software, Campbell, Australia) but fitted using both Scrubber and CLAMP software (23).

The data were fitted using a mass transport model (24, 25). Three independent surfaces were globally fit for the association and dissociation rates *k_a_* and *k_a_*, while floating the maximum binding response (*R*_max_) and mass transport (*k_t_*) constant for each surface. The solutions from the fits for all three surfaces from both Scrubber and CLAMP all showed k_a_*R_max_/*k_t_* values of <5 (a measure of the suitability of the solution to yield unique values for *k_a_* and *k_d_*) (26). Additionally, kinetic values derived from the global fit using CLAMP software were not highly correlated. The observed correlation coefficients for *k_a_ versus k_d_* were 0.62 and 0.67 in replicate experiments, demonstrating that although the sensorgrams are influenced by mass transport, a unique solution for these parameters can be determined (24, 25). Both fitting routines yielded values for *k_a_* and *k_d_* for identical surfaces that did not differ appreciably (< 4.5%). For wortmannin, only an off-rate analysis was performed.

##### X-ray Crystallography

Crystals used for seeding were obtained by vapor diffusion at 20 °C with 25 nl of 11 mg/ml ΔABD-p110δ and 0.9 mm of a p110δ inhibitor in protein storage buffer (20 mm Tris, pH 7.2, 50 mm (NH_4_)_2_SO_4_, 1% v/v ethylene glycol, 1% (w/v) betaine, 0.3 μm CHAPS, and 5 mm TCEP) added to 25 nl of reservoir solution (25% (w/v) PEG 3350, 0.1 m bis-tris (pH 6.5). Crystals were pulverized, pooled together in the reservoir solution, vortexed with a Seed-Bead (Hampton Research) for 45 s, flash-frozen in liquid nitrogen, and stored at −80 °C. For seeding crystallization trials, the frozen seed was thawed and diluted 500-fold in 25% (v/v) PEG 300, 0.1 m Tris (pH 8.5).

Preparation of diffraction quality ΔABD-p110δ:idelalisib crystals started with a mixture of 0.48 μl of 2.5% (w/v) *n*-dodecyl-β-d-maltoside, 0.30 μl of 20 mm ligand, and 12 μl of 12 mg/ml ΔABD-p110δ in storage buffer (described above). The mixture was allowed to sit for 1 h at room temperature instead of 4 °C to prevent the formation of white precipitate. Seeded vapor diffusion droplets were assembled by adding 90 nl of the 500-fold diluted seed (described above) to 100 nl of the *n*-dodecyl-β-d-maltoside-ligand-ΔABD-p110δ mixture. The droplets were equilibrated against reservoir wells containing 50 μl of 1% to 30% (v/v) PEG 300, 0.1 m Tris (pH 8.5) at 20 °C. Crystals appeared in 2–5 days across the 1% to 30% PEG range. Crystals were cryoprotected in 20% (w/v) glycerol, 25% (w/v) PEG 300, 0.1 m Tris (pH 8.5), 50 mm ammonium sulfate, 0.2% (w/v) *n*-dodecyl-β-d-maltoside, 0.2 mm ligand and were flash-frozen in liquid nitrogen for data collection.

Diffraction data were collected on Beamline 5.0.1 at the Advanced Light Source (Berkeley, CA). The data were reduced using Mosflm (27) and Aimless (28). The structure was solved by molecular replacement using EPMR (29) and refined using the Phenix software package (30), and the electron density was fit using Coot (31). The inhibitor and surrounding ATP-binding site are very clearly defined in the electron density maps. The coordinates as well as the diffraction data have been deposited in the Protein Data Bank under code 4XE0.

## RESULTS AND DISCUSSION

### 

#### 

##### In Vitro Potency and Selectivity

Table 1 shows the idelalisib IC_50_ values for each class I isoform measured in the presence of 2 × *K_m_* ATP. The IC_50_ of idelalisib for PI3Kδ is 19 nm, whereas the IC_50_ values for PI3Kα, PI3Kβ, and PI3Kγ are 8,600, 4,000, and 2,100 nm, respectively. Thus, idelalisib potently inhibits PI3Kδ and is selective relative to the other class I isoforms (453-, 210-, and 110-fold selective against the α, β, and γ isoforms, respectively). Testing of a broad panel of 351 kinases and 44 mutant kinases (21) at 10 μm idelalisib showed that the most potent inhibition seen for any non-PI3K kinase was a percent inhibition of 47%.

**TABLE 1:** **Idelalisib potency and class I selectivity at [ATP] = 2 × *K_m_***

	Time-resolved FRET ATP *K_m_*	IC_50_ at 2 × *K_m_* ATP	IC_50_-based PI3Kδ fold selectivity
	μ*m*	*nm*	
PI3Kα	48	8600	453
PI3Kβ	279	4000	210
PI3Kδ	118	19	1
PI3Kγ	37	2100	110

##### Idelalisib Is an ATP-competitive Inhibitor

**FIGURE 2.**
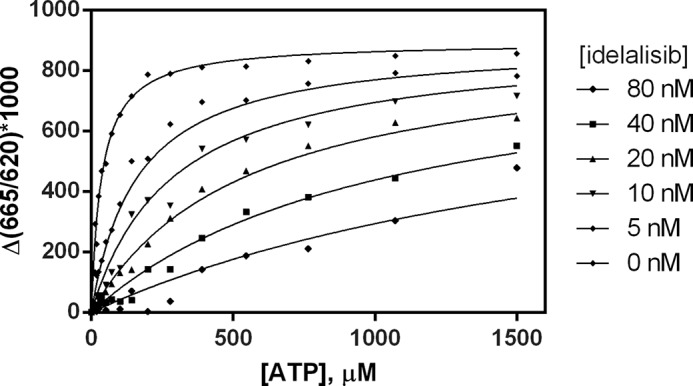
**Competition of idelalisib with ATP against PI3Kδ.** Reaction rates were determined at various concentrations of ATP and idelalisib, and the data were fit globally with a general inhibition model to characterize the competitive behavior.

The ability of idelalisib to compete with ATP binding to PI3Kδ was tested in enzymatic assays. The activity of PI3Kδ was measured at various concentrations of ATP and idelalisib, and the data were fit globally to the competition expression described under “Experimental Procedures.” The results are shown in Fig. 2. The global fit yielded the following kinetic parameters: *K_m_* = 37 ± 3 μm and *K_i_* = 1.5 ± 0.1 nm. The measured *K_m_* value of 37 μm from the competition data was slightly lower than the value reported in Table 1, but this difference did not significantly affect the data analysis.

The fit for the parameter α gave a very high value that reached the limits of the data fitting program. The high value of α (α ≫ 1) demonstrates ATP-competitive inhibition by idelalisib. As confirmation of the ATP competition, a fit of the kinetic data with a pure competitive model gave identical values for *K_m_* and *K_i_* with the same goodness of fit (data not shown).

##### Idelalisib Is a Reversible Inhibitor

**FIGURE 3.**
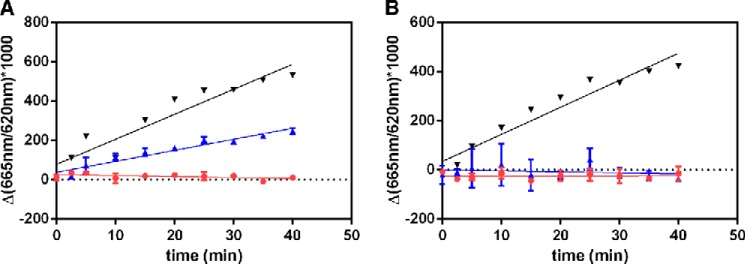
**Reversibility experiments with idelalisib (*A*) and wortmannin (*B*).** For each experiment, the progress curves are denoted as follows: *black*, DMSO (no inhibitor) enzyme control; *blue*, activity following 100-fold dilution of test compound preincubated with enzyme; and *red*, enzyme activity in the presence of 400 nm idelalisib or 100 nm wortmannin. The *red progress curve* gives the expected activity of the preformed enzyme-inhibitor complex if no compound dissociation occurs upon dilution.

Along with the kinetic characterization of idelalisib described above, two additional studies showed that it is a reversible inhibitor. Dilution experiments showed that the kinase can be completely inhibited with idelalisib at concentrations ∼20-fold greater than its IC_50_. However, enzyme activity was quickly recovered following a 1:100 dilution of the enzyme-inhibitor complex into a buffer solution containing the ATP and PIP2 substrates. DMSO and the irreversible inhibitor wortmannin were used as control compounds (Fig. 3, *A* and *B*). The rate of PIP2 phosphorylation upon dilution was approximately one-half of that of the DMSO control. This rate is consistent with the steady-state reaction rate of PI3Kδ in the presence of 4 nm idelalisib. Because the reaction was sampled at 5-min intervals, no distinct kinetic phase for compound dissociation was observed. However, based on the linearity of the progress curve, the dissociation of inhibitor likely occurred within the first few minutes of reaction time. In contrast, enzyme preincubated with wortmannin did not show detectable recovery of activity upon dilution. The dilution of wortmannin-inhibited PI3Kδ did not lead to an increased reaction rate, implying that no dissociation of wortmannin occurred during dilution and the subsequent enzymatic reaction. These results demonstrate that idelalisib is a reversible inhibitor of PI3Kδ.

**FIGURE 4.**
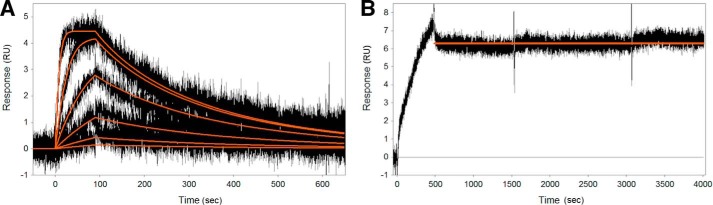
**Shown are sensorgrams from representative surfaces of neutravidin-captured p110δ binding to idelalisib (*A*) and wortmannin (*B*).** Idelalisib was injected at concentrations of 0.12, 0.37, 1.1, 3.3, 10, and 30 nm (the latter being injected in duplicate). Wortmannin was injected at a concentration of 2 μm. *RU*, response units.

Surface plasmon resonance studies further confirmed the reversibility of idelalisib. The Biacore platform was used to examine the direct molecular interaction of idelalisib to biotinylated p110δ captured on a neutravidin surface. Sensorgrams for idelalisib (Fig. 4*A*) showed a specific, saturable, dose-responsive interaction with p110δ. Duplicate injections of the inhibitor at 30 nm on the same surface overlay well, demonstrating the stability of the captured protein. The maximum signal, *R*_max_, was kept low at single-digit response units to minimize the influence of mass transport for this compound. Idelalisib binding is characterized by a very fast on rate, *k_a_* = 5.18 × 10^6^ m^−1^ s^−1^, and a moderate off rate, *k_d_* = 6.34 × 10^−3^ s^−1^. A replicate experiment yielded values that differed by 3 and 16% in *k_a_* and *k_d_*, respectively. More importantly, the response signal during the dissociation phase returned to baseline level for idelalisib, indicating complete dissociation of the compound from the protein. The wortmannin control (Fig. 4*B*) showed no dissociation after 1 h, consistent with this compound binding irreversibly to p110δ (32). No nonspecific binding of idelalisib and wortmannin on the neutravidin reference surface was observed (data not shown). Biotinylation without idelalisib protection did not affect the results (data not shown). The reversibility of idelalisib may have implications for drug safety; some irreversible inhibitors may be more susceptible to off-target binding or have a slightly increased risk of idiopathic toxicity compared with reversible inhibitors (33).

##### Crystal Structure of PI3Kδ in Complex with Idelalisib

**FIGURE 5.**
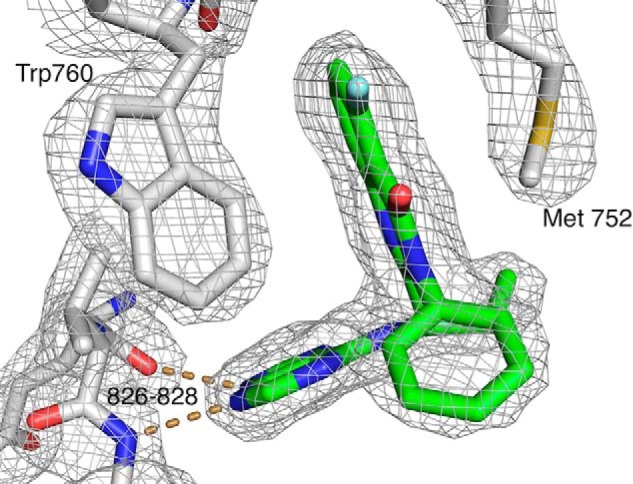
**X-ray crystal structure of idelalisib bound in the PI3Kδ ATP-binding site.** The 2*F*_o_ − *F*_c_ electron density is contoured at 1.2 σ. The *dashed lines* represent hydrogen bonds to the hinge region of the kinase. For clarity, only a few residues are shown.

We determined the crystal structure of ΔABD-p110δ in the presence of idelalisib (Fig. 5) (Protein Data Bank code 4XE0). The data and refinement statistics are summarized in Table 2.

**TABLE 2:** **Data and refinement statistics**

**Data**	
Space group	C2
Cell parameters (Å)	142.1, 64.6, 116.0, β = 103.1°
Resolution range (last shell) (Å)	69.2–2.4 (2.5–2.4)
Number of observations	136,588
Number of unique reflections	38,471
Completeness (last shell) (%)	99.4 (98.8)
<*I*>/<σ(*I*)> (last shell)	9.7 (2.7)
CC1/2 (last shell)	0.995 (0.77)
Average B-factor of the model (A^2^)	41
Wilson B-factor (Å^2^)	36.0

**Model**	
Subunits in asymmetric unit	1
Number of water molecules	224
Resolution used for refinement (Å)	69.2-2.4
Sigma cutoff (*F*/σ(*F*))	0.0
R-factor (last shell)	0.21 (0.24)
Free R (last shell)	0.29 (0.33)
Root mean square deviation from ideal geometry: bonds (Å)/angles (°)	0.009/1.21
Ramachandran plot: favored/outliers (%)	94/1.5

The protein used for crystallography was comprised of 939 residues, of which 815 were observed in the electron density. Aside from the missing residues, a significant fraction of the protein has weak electron density and correspondingly high temperature factors. The kinase domain and, in particular the ATP-binding site, is well defined by the electron density, but the Ras-binding and C2 domains have higher temperature factors and are more mobile, as are the residues that link the ABD and the Ras-binding domain. The C-terminal end of the helical domain (roughly residues 630–675) interacts with residues from both the N- and C-terminal lobes of the kinase. This section shows relatively low temperature factors, whereas the rest of the helical domain is more mobile. It seems likely that the more mobile parts of the protein would be stabilized when interacting with the ABD, the p85 subunit, and with additional binding partners in the cell.

**FIGURE 6.**
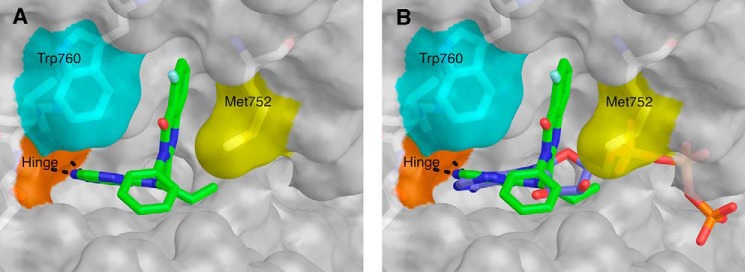
*A*, structure of idelalisib (*green*) bound to the PI3Kδ ATP-binding site. Met-752 (*yellow*) and Trp-760 (cyan) form the sides of the specificity pocket. The hinge region is shown in *orange*, and hydrogen bonds between the inhibitor and the hinge are shown as *dashed lines. B*, idelalisib (*green*) bound to PI3Kδ. The ATP was placed in the PI3Kδ binding site by superimposing (using the “align” command in PyMOL) the structure of PI3Kγ bound with ATP (accession code 1E8X) (35).

Idelalisib binds in the ATP-binding pocket (Fig. 6, *A* and *B*). When compared with the binding of ATP, the inhibitor forms hydrogen bonds to the hinge region that are very similar to those made by ATP (Fig. 6*B*). However, the remaining interactions with the protein are very different from what are seen for ATP binding (Fig. 6*B*).

Idelalisib is a propeller-shaped inhibitor and adopts a binding mode similar to what was described for the binding of another propeller-shaped inhibitor: IC87114 (20, 34). The electron density for idelalisib shows that there is no covalent bond between the inhibitor and the protein. This is in contrast, for example, with the electron density of wortmannin bound to PI3Kγ, where there is clear density for the covalent bond with Lys-833 (35).

**FIGURE 7.**
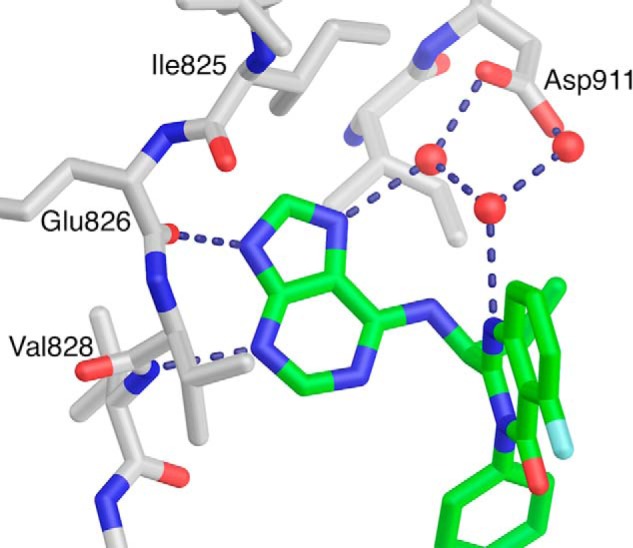
**Water-mediated hydrogen bonds between the p110δ and idelalisib.** To expose the inhibitor, much of the protein is not shown.

The purine group of idelalisib serves as the hinge binder, with hydrogen bonds between the purine N3 and the backbone amide of Val-828 and between N9 and the backbone carbonyl oxygen of Glu-826 (Fig. 7). The purine N7 forms part of a water-mediated hydrogen bond network that includes N1 of the quinazolinone and the side chain of Asp-911 (Fig. 7). The ethyl group of the inhibitor binds in a hydrophobic pocket formed by Ile-910, Met-900, and Met-752.

**FIGURE 8.**
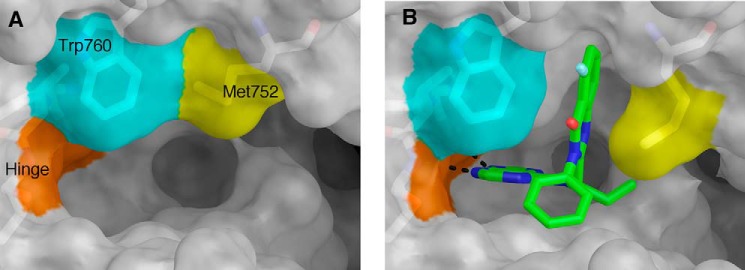
**Comparison of apo PI3Kδ (Protein Data Bank accession code 2WXR) (*A*) and idelalisib-bound PI3Kδ showing the opening of the specificity pocket that accompanies idelalisib binding (*B*).**

A comparison of the idelalisib-bound and apo structures (Protein Data Bank code 1EAX) shows that the ATP-binding site requires a substantial conformational change to accommodate idelalisib (Fig. 8, *A* and *B*). In the apo enzyme, Met-752 and Trp-760 are packed against each other, but in the presence of idelalisib these two residues lie ∼6.5 Å apart, opening a hydrophobic pocket (the specificity pocket) in which the fluoro-quinazolinone of the inhibitor binds (Fig. 8, *A* and *B*). Similar to what was seen previously, the plane of the quinazolinone ring system is parallel to the sides of the pocket, displaying a hydrophobic interaction with the kinase (20).

The phenyl moiety of idelalisib packs against the side chains of Asp-832, Thr-833, and Asn-836. Our SAR data^3^ and the work of Cushing *et al.* (36) on another propeller-shaped series suggest that modifications at this position affect the potency and selectivity across the PI3K class I family.

The p110δ side chains that interact with idelalisib are conserved across the class I isoforms. However, the opening of the specificity pocket requires conformational changes in areas further from the ATP-binding site that no longer share sequence identity across the family. Knight *et al.* (37) studied the binding of PI3Kγ to another propeller-shaped inhibitor (PIK-39) and made the argument that the selectivity of the propeller-shaped inhibitors is at least partially a reflection of how much energy it takes to open the specificity pocket in each isoform. Berndt *et al.* (20) came to the same conclusion based on their structural and modeling work on IC87114 binding to PI3Kδ. IC87114 has a purine hinge binder and accesses the specificity pocket with a quinazolinone. Overall, these observations support the idea that engagement of the specificity pocket upon kinase binding contributes to the selectivity profile of idelalisib.

##### Conclusions

Our *in vitro* biochemical data showed that idelalisib potently and selectively inhibits the kinase activity of PI3Kδ, and the kinetic characterization of inhibition demonstrated that it is competitive with ATP. The kinetic analysis, along with the dilution and surface plasmon resonance experiments, clearly showed that idelalisib binds reversibly to the enzyme.

The 2.4 Å crystal structure of the idelalisib-PI3Kδ complex showed the inhibitor binding in the ATP-binding site and revealed the specific and entirely noncovalent interactions between inhibitor and protein. Idelalisib binds so as to open and occupy the specificity pocket, as seen for other propeller-shaped inhibitors. More generally, the structure provides a framework for understanding the potency of idelalisib, as well as its selectivity for PI3Kδ over the rest of the class I PI3Ks and across the kinome.

## Footnotes

3 Manuscript in preparation.

## Footnotes

2 The abbreviations used are:

PIP2: phosphatidylinositol 4,5-bisphosphate
PIP3: phosphotidylinositol 3,4,5-trisphosphate
Ni-NTA: nickel-nitrilotriacetic acid
ABD: adaptor-binding domain
bis-tris: 2-[bis(2-hydroxyethyl)amino]-2-(hydroxymethyl)propane-1,3-diol
TCEP: tris(2-carboxyethyl)phosphine hydrochloride.
